# Immunotherapy Advances in Locally Advanced and Recurrent/Metastatic Head and Neck Squamous Cell Carcinoma and Its Relationship With Human Papillomavirus

**DOI:** 10.3389/fimmu.2021.652054

**Published:** 2021-07-08

**Authors:** Huanhuan Wang, Qin Zhao, Yuyu Zhang, Qihe Zhang, Zhuangzhuang Zheng, Shiyu Liu, Zijing Liu, Lingbin Meng, Ying Xin, Xin Jiang

**Affiliations:** ^1^ Department of Radiation Oncology, The First Hospital of Jilin University, Changchun, China; ^2^ Jilin Provincial Key Laboratory of Radiation Oncology & Therapy, The First Hospital of Jilin University, Changchun, China; ^3^ National Health Commission (NHC) Key Laboratory of Radiobiology, School of Public Health, Jilin University, Changchun, China; ^4^ Key Laboratory of Pathobiology, Ministry of Education, Jilin University, Changchun, China; ^5^ Department of Hematology and Medical Oncology, Moffitt Cancer Center, Tampa, FL, United States

**Keywords:** head and neck squamous cell carcinoma, immunotherapy, human papillomavirus, immune-checkpoint inhibitors, immune characteristics

## Abstract

Head and neck cancer (HNC) is the sixth most common malignancy worldwide; head and neck squamous cell carcinoma (HNSCC) account for the most cases of HNC. Past smoking and alcohol consumption are common risk factors of HNSCC; however, an increasing number of cases associated with human papillomavirus (HPV) infection have been reported in recent years. The treatment of HNSCC is integrated and multimodal including traditional surgery, radiotherapy, chemotherapy, and targeted therapy. Since pembrolizumab was approved in 2016, an increasing number of studies have focused on immunotherapy. However, not all of HNSCC patients have a better outcome on immunotherapy. Immunotherapy has been reported to be more effective in HPV-positive patients, but its molecular mechanism is still unclear. Some researchers have proposed that the high proportion of infiltrating immune cells in HPV-positive tumors and the difference in immune checkpoint expression level may be the reasons for their better response. As a result, a series of individualized immunotherapy trials have also been conducted in HPV-positive patients. This paper summarizes the current status of HNSCC immunotherapy, individualized immunotherapy in HPV-positive patients, and immune differences in HPV-positive tumors to provide new insights into HNSCC immunotherapy and try to identify patients who may benefit from immunotherapy.

## Introduction

Head and neck cancer (HNC) is the sixth most common malignancy worldwide, with approximately 800,000 new cases and 400,000 deaths annually (1), of which head and neck squamous cell carcinoma (HNSCC) account for >90% cases. Past smoking and alcohol consumption are major risk factors of HNSCC. However, the incidence of human papillomavirus (HPV)-associated HNSCC has been increasing recently (2, 3), especially HPV-associated oropharyngeal squamous cell carcinoma (OPSCC) (4, 5). Of the >200 subtypes of HPV have been identified (6), HPV16 is the most closely related to the occurrence and development of HNSCC (7). The prevalence of HPV16 is >80% among HPV-infected OPSCC patients (4, 8). E6 and E7, the two major oncogenic proteins of HPV, can downregulate the tumor suppressor factors TP53 and RB (9, 10), thereby mediating the expression cytokines, leading to immune escape (11), downregulating the interferon pathway, and resulting in an immune-privileged tumor state (12). The above mechanism is the main mechanism underlying HPV-driven HNSCC. Although HPV-positive tumors are more advanced, have a greater burden of disease, but patients with such tumors generally have higher survival rates (13–15), possibly because they have a better treatment response (16).

The treatment of HNSCC is integrated and multimodal including surgery, radiotherapy, chemotherapy and so on (17–19). Patients with early-stage tumors are often considered curable. However, most patients have the advanced disease, often involving the lymph nodes (20, 21). The overall survival rates of patients with advanced disease remain low and most patients relapse within 3–5 years, despite the use of a platinum-based chemotherapy of treatments (22). Nevertheless, combination therapy has been the mainstay of treatment for decades until 2016, when a new immunotherapy was introduced, ushering a new era for HNSCC therapy. Programmed death receptor-1 (PD-1) inhibitors were approved in 2016 for recurrent/metastatic (R/M) HNSCC that progresses after chemotherapy failure. Subsequently, studies on immune-checkpoint inhibitors (ICs) against PD-1, programmed death receptor ligand-1 (PD-L1), and cytotoxic T lymphocyte-associated protein-4 (CTLA-4) were performed (23–25). To date, five PD-1 inhibitors for HNSCC have been developed to the second line of therapy, with the most comprehensive data available for pembrolizumab (KEYNOTE-012 trial) and nivolumab (CheckMate-141 trial) (26, 27). Currently, these two PD-1 inhibitors have been approved by the Food and Drug Administration (FDA) for the treatment of platinum-resistant HNSCC (26, 27). ICs have achieved good efficacy in some HNSCC, indicating that targeted immune system therapy can achieve clinical benefits in HNSCC patients (28, 29). However, most patients show primary resistance, and it is unclear which patients with HNSCC will benefit the most from immunotherapy (30). Studies have shown that immune differences in HPV-positive HNSCC patients may make immunotherapy more effective (29). Hence, studies on immunotherapy in HPV-positive HNSCC have also been conducted.

This article aimed to review the progress of immunotherapy for HNSCC, and to further elucidate the differences between HPV-positive and HPV-negative of immune levels, and the molecular mechanisms underlying the different immunotherapy effects.

## Immunotherapy in HNSCC

**Figure 1:**
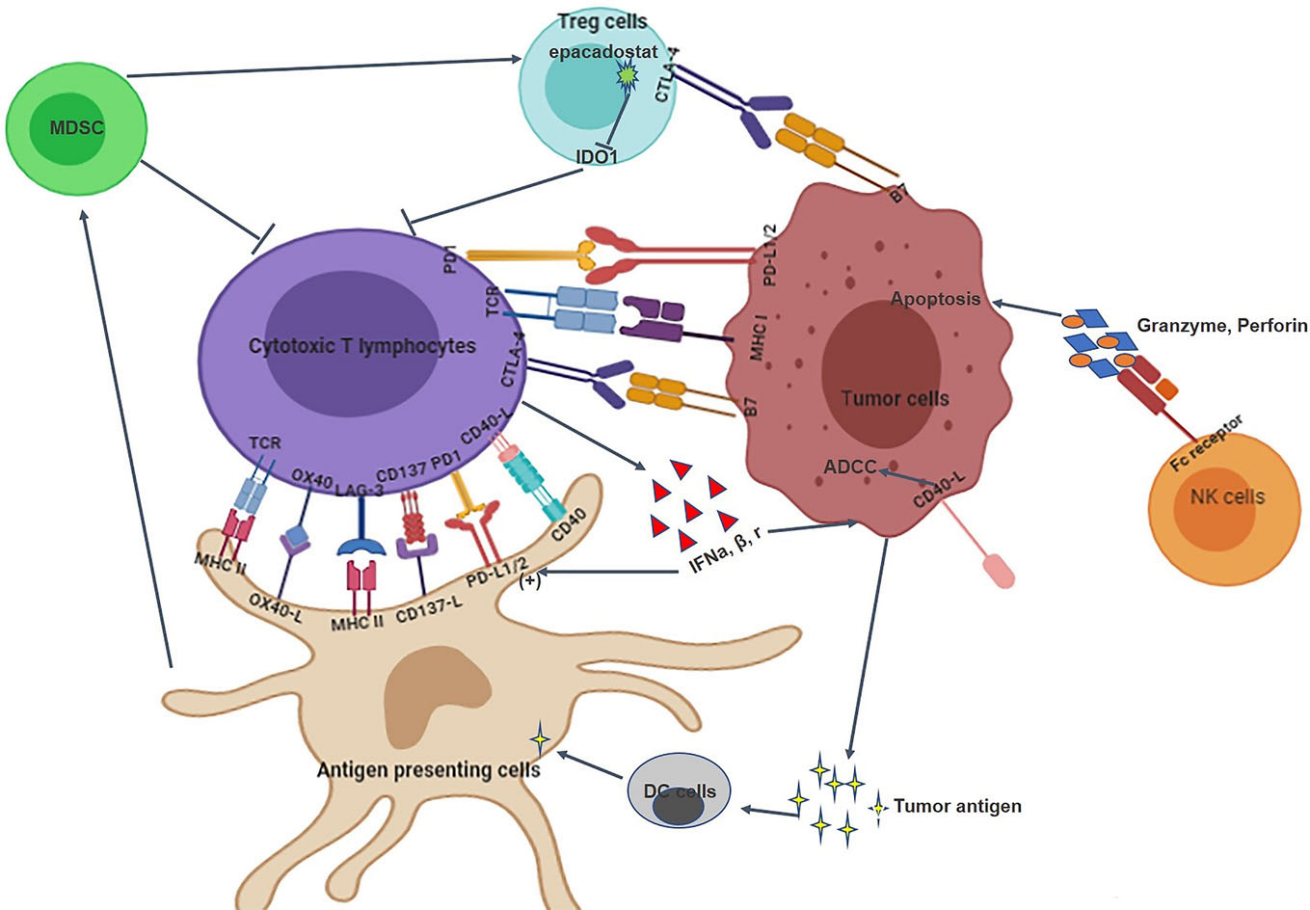
The interaction between HNSCC tumor cells and the immune system. 1) The immune system of HNSCC tumor tissues includes immune cells, immune checkpoints, and immune microenvironment; 2). Immune checkpoints such as PD-1/PD-L1 and CTLA-4/B7 can inhibit the proliferation and infiltration of cytotoxic T lymphocytes; 3) MDSC, Treg cells, NK cells and cytotoxic T lymphocytes are involved in the elimination of tumor cells; 4) Tumor cells and immune cells can also interact with each other through cytokines.

The Cancer Genome Atlas (TCGA) data shows that HNSCC is the most immune-active tumor tissue after lung adenocarcinoma and renal cell carcinoma (31–33). The occurrence and progression of HNSCC is associated with serious immune deficiency, including immune cell dysfunction, decreased cytokine secretion, and antigen presentation defects (34, 35). Therefore, targeting the immune system is expected to become a new treatment strategy for HNSCC (36). The interactions between tumor cells and the immune system in HNSCC is shown in **Figure 1**. This study aimed to review immunotherapy for HNSCC from these aspects: immune checkpoint and immune microenvironment.

Immune checkpoints are part of the protein-ligand receptor system that controls T cell activation. Therefore, the application of ICs to block the role of immune checkpoints can promote the release of T cells, increase the antitumor response, and, thus, enhance tumor cell clearance and immune monitoring. PD-1 is a transmembrane protein of the CD28 family of T cell costimulatory receptors, which is expressed in a variety of immune cells, especially in cytotoxic T cells (37, 38). PD-1 binds to its ligands (PD-L1 and PD-L2), reducing T cell activity and maintaining immune tolerance (39). Therefore, the binding of monoclonal antibodies to PD-1 or PD-L1 can block the inhibitory function of immune checkpoints on T cells to restore the T cell immune response. Pembrolizumab (KEYNOTE-012 trial), the first reported PD-1 inhibitor for R/M HNSCC, had a response rate of 18% (26). This trial supported further study of pembrolizumab as anticancer therapy for HNSCC. Subsequently, the KEYNOTE-055 trial also confirmed this finding (40). Based on these findings, pembrolizumab received accelerated FDA approval in 2016 for the treatment of HNSCC. The KEYNOTE-040 was the randomized, open-label, phase III study, which included 495 patients with refractory HNSCC. The results showed that the median survival time in the pembrolizumab and standard treatment groups was 8.4 and 6.9 months, respectively. In addition, pembrolizumab group had fewer treatment-related adverse events of grade 3 and worse than standard treatment group (30). The results suggest that pembrolizumab may be monotherapy and a part of combination therapy for HNSCC. Subsequently, a clinical trial using pembrolizumab in combination with chemotherapy was conducted. The KEYNOTE-048 trial compared the effectiveness of pembrolizumab alone and pembrolizumab combined with chemotherapy to traditional chemotherapy in patients who relapsed 6 months after standard treatment, which results showed pembrolizumab with chemotherapy improved overall survival *versus* chemotherapy (13 months *vs*. 10.7 months, HR 0.77, p=0.0034) (28). Thus, pembrolizumab alone and pembrolizumab in combination with platinum and 5-fluorouracil can be used as an appropriate first-line treatment for R/M HNSCC. In view of this, more clinical trials are currently underway, including a comparison of pembrolizumab plus radiotherapy with chemotherapy (NCT02641093). In addition, studies have compared the efficacy of pembrolizumab and the oncolytic virus Talimogene laherparepvec (NCT02626000) and the use of pembrolizumab in combination with other agents such as colony-stimulating factor receptor kinase inhibitors (NCT02452424) and histone deacetylase inhibitors (NCT02538510).

CheckMate 141 was the first reported phase III clinical trial of a PD-1 inhibitor, which was designed to compare the efficacy of nivolumab to that the conventional regimen in R/M HNSCC (27). The results showed that nivolumab was significantly better than traditional chemotherapy, with an increased median survival time of 2.4 months (7.5 months *vs*. 5.1 months), a 20% higher 1-year survival rate (36% *vs*. 16%), and a significantly reduced risk of severe adverse reactions (27). Hence, FDA approved nivolumab as a second-line treatment for R/M HNSCC in 2016. In addition, nivolumab in combination with lirilumab, an NK cell-targeted antibody, showed an objective response rate of 24%, and a tumor load reduction of 80% in patients with HNSCC (41, 42). In NCT02426892, nivolumab in combination with the HPV vaccine (ISA101) showed significant activation of T cells and better anti-tumor response in patients with OPSCC (43). In addition, ongoing clinical studies have been assessing the effectiveness of nivolumab in combination with other regimens, such as nivolumab combined with stereotactic radiotherapy (SBRT) (NCT02684253).

Clinical trials of PD-L1 antibodies are also widely underway, such as durvalumab, atezolizumab, and avelumab. A phase II clinical study showed a 16% response rate and a 33% 1-year survival rate for 100 patients administered durvalumab (44). In addition, several clinical studies of durvalumab monotherapy are ongoing (such as NCT02207530 and NCT02827838). The phase I clinical study of atezolizumab showed an effective rate of 22% (45).The phase I clinical trial that evaluate the efficacy of avelumab and CD137 agonists (NCT02554812) is underway.

In addition to PD-1 and PD-L1 inhibitors being applied on HNSCC, CTLA-4 is also an important immune checkpoint. Tremelimumab is an antibody to CTLA-4, CONDOR and EAGLE trial concluded that durvalumab alone or in combination with tremelimumab in patients with R/M HNSCC does not show a significant difference in efficacy (46, 47). Ipilimumab is also a monoclonal antibody against CTLA-4. Clinical trials of ipilimumab and cetuximab combined with IMRT in patients with advanced HNSCC are ongoing (NCT01860430 and NCT01935921). In addition, clinical trials of CD276 and OX40 as new immunotherapeutic targets are also being carried out widely, such as NCT02381314, NCT02274155, etc.

In addition, other immunotherapy drugs have been studied successively, such as the indoleamine 2,3-dioxygenase 1 (IDO1) inhibitors navoximod and epacadostat. IDO1, a rate-limiting enzyme converted to kynurenine by tryptophan, plays an immunosuppressive role in tumor immune microenvironments (48, 49). In previous studies, upregulation of IDO1 inhibited the function of antitumor T cells (50) and high IDO1 expression was associated with poor prognosis (51). IDO1 inhibitor can restore T lymphocyte function, resulting in tumor microenvironment immunogenicity (52). In a phase I clinical trial, navoximod showed good efficacy in 36% patients (53). KEYNOTE-037 has also reported that epacadostat combined with pembrolizumab has good antitumor effects (54).

Research on immunotherapy for small molecule receptor agonists such as immunoglobulin 2 (IgG2) CD137 agonists, and toll-like receptor 8 (TLR8) agonists are underway. Such as, IgG2 CD137 agonists combined with nivolumab for patients with advanced HNSCC (e.g., NCT02253992). TLR8 agonists stimulate the immune system, further blocking tumor cell survival. NCT01334177 was a phase Ib clinical study designed to evaluate the efficacy of the TLR8 agonist VTX-2337 combined with cetuximab in the treatment of R/M HNSCC. The results showed an objective response rate of 15% and a disease control rate of 54%, with no serious toxicity or deaths; thus, the TLR8 agonist combined with cetuximab was proved to safe and effective. In NCT01836029, a phase II clinical study, patients with HNSCC also showed good tolerance to VTX-2337 combined with standard chemotherapy; however, significant differences were observed in the overall and progression-free survival (55). A further phase II clinical study of VTX-2337 in combination with nivolumab is ongoing (NCT02124850). In addition, research is being conducted on antitumor vaccines. NCT01998542, a phase II clinical trial completed in 2020, has compared the efficacy and safety of the personalized antitumor vaccine AlloVax (^™^) for the treatment of R/M HNSCC; the results are awaited. All of ongoing and completed immunotherapy trials are summarized in **Tables 1** and **2**, respectively.

**Table 1:** Summary of ongoing clinical trials in HNSCC.

ID	Phase	N	Inclusion criteria	Interventions	Immunotherapy targets	Primary Outcome
NCT03406247	II	140	R/M HNSCC	Nivolumab *vs* Nivolumab + Ipilimumab	PD-1, CTLA-4	2-years DFS
NCT04326257	II	40	R/M HNSCC	Nivolumab + Relatlimab *vs* Nivolumab + Ipilimumab	PD-1, LAG-3, CTLA-4	ORR
NCT04428151	II	400	R/M HNSCC	Lenvatinib + Pembrolizumab *vs* Chemotherapy + Lenvatinib	PD-1	ORR
NCT02296684	II	66	Locoregionally Advanced, Resectable HNSCC	Neoadjuvant Pembrolizumab *vs* Adjuvant Pembrolizumab	PD-1	LRR, Distant failure rate
NCT03283605	I/II	45	Metastatic HNSCC	Durvalumab, Tremelimumab + SBRT	PD-L1, CTLA-4	Acute toxicities, PFS
NCT03620123	II	280	R/M HNSCC	Nivolumab and Ipilimumab *vs* Docetaxel	PD-1, CTLA-4	ORR
NCT03313804	II	57	Advanced, Metastatic HNSCC	Nivolumab OR Pembrolizumab OR Atezolizumab + RT	PD-1, PD-1, PD-L1	PFS
NCT01149902	I	10	Relapsed, Refractory HNSCC	cyclophosphamide, docetaxel, OK-432	Vaccine	Safety and Feasibility
NCT03341936	II	58	Relapsed, Resectable HNSCC	Neodjuvant Nivolumab, Lirilumab + Surgery + Adjuvant Nivolumab, Lirilumab	PD-1, KIR	DFS
NCT03098160	I	69	Metastatic, Locally Advanced, HPV- HNSCC	Evofosfamide,,Ipilimumab	CTLA-4	RP2D
NCT03317327	I/II	20	Recurrent, secondary primary HNSCC	Nivolumab + RT	PD-1	Incidence, Nature, and Severity of AE
NCT02999646	II	41	Stage III/IV, R/M HNSCC	MVX-ONCO-1	Autologous Tumor Cells	OS
NCT04183166	I	30	Stage III/IVA HNSCC	TG4050	Vaccine	Safety and Tolerability
NCT03708224	II	60	HPV-, Stage III/IV HNSCC	Atezolizumab *vs* Atezolizumab + Immune-modulating Agent	PD-L1	R0 resection rate, CD3 counts
NCT04247282	I/II	40	Untreated intermediate/high risk, p16-negative HNSCC	M7824 *vs* M7824 + TriAd vaccine *vs* M7824 + TriAd vaccine + N-803	PD-L1, Vaccine,	pCR
NCT03695510	II	29	R/M HNSCC	Afatinib + Pembrolizumab	PD-1	ORR
NCT03552718	I	16	HNSCC	YE-NEO-001	Vaccine	Incidence of AE, RP2D
NCT03088059	II	340	R/M HNSCC	Afatinib, Palbociclib, Niraparib, BAY1163877, IPH2201, Durvalumab	NKG2A, PD-L1	PFS, ORR
NCT03129061	I	24	Unresectable, Metastatic HNSCC	baseline + Nivolumab, Pembrolizumab	PD-1	T cell activation
NCT03522584	I/II	20	R/M HNSCC	Tremelimumab, Durvalumab, HIGRT, SBRT	CTLA-4, PD-L1	Incidence of AE
NCT04220775	I/II	21	Local-regional Recurrent HNSCC	M7824, SBRT	PD-L1	PFS
NCT03975270	II	41	R/M HNSCC	Sintilimab + Nab-paclitaxe	PD-1,	ORR
NCT04139057	I/II	9	EBV +,R/M HNSCC,	EBV-specific TCR-T cells	Engineered T cells	MTD
NCT04193293	I/II	30	R/M HNSCC	Duvelisib + Pembrolizumab	PD-1	Rate of DLT, AE, ORR
NCT03823131	II	68	Unresectable, R/M HNSCC	Tavo-EP, Pembrolizumab, Epacadostat	PD-1, IDO1	ORR
NCT02997332	I	36	Locally Advanced, untreated HNSCC	Durvalumab, Docetaxel, Cisplatin, 5-FU	PD-L1,	PR2D, Number of DLT
NCT03546582	II	102	Locoregionally Recurrent or Second Primary HNSCC	Pembrolizumab + SBRT *vs* SBRT	PD-1	PFS
NCT03565783	II	44	Advanced-Stage, Resectable HNSCC	Cemiplimab	PD-1	Overall response rate
NCT03548467	I/II	65	Locally advanced, Metastatic HNSCC	VB10.NEO + Bempegaldesleukin	Vaccine	Rate of AE
NCT03245489	I	20	R/M HNSCC	Pembrolizumab + Clopidogrel + Acetylsalicylic acid *vs* Pembrolizumab	PD-1	Effect on major cellular parameters
NCT04282109	II	141	R/M HNSCC	Nivolumab + Paclitaxel *vs* Cetuximab + Paclitaxel	PD-1	OS
NCT03426657	II	120	Locally Advanced HNSCC	Durvalumab + Tremelimumab + RT	PD-L1, CTLA-4	PFS
NCT03529422	II	33	Intermediate Risk HNSCC	Durvalumab + IMRT	PD-L1	DFS
NCT04357873	II	111	R/M HNSCC	Pembrolizumab + Vorinostat	PD-1	ORR
NCT03629756	I	44	Advanced, Recurrent HNSCC	AB928, AB122	PD-1	Rate of AE and DLT
NCT02812524	I	18	HNSCC	Intratumoral Ipilimumab	CTLA-4	Surgery delay rate
NCT02764593	I	40	Intermediate/High-Risk Local-Regionally Advanced HNSCC	Nivolumab + Cisplatin, Nivolumab + High-dose Cisplatin, Nivolumab + Cetuximab, Nivolumab + IMRT	PD-1	DLT
NCT03226756	II	351	R/M HNSCC	Nivolumab	PD-1	Incidence for AEI
NCT04107103	II	20	Recurrence HNSCC	Nivolumab + Pemetrexed	PD-1	Feasibility, Safety/tolerability
NCT03085719	II	26	R/M HNSCC	High Dose Radiation + Pembrolizumab *vs* High Dose + Low Dose Radiation + Pembrolizumab	PD-1	Overall Response Rate
NCT03854032	II	48	Stage II-IV HNSCC	BMS986205 + Nivolumab *vs* Nivolumab	IDO1, PD-1	ORR
NCT02999087	III	688	Locally Advanced HNSCC	Cisplatin + IMRT *vs* Cetuximab + Avelumab + IMRT *vs* Cetuximab + IMRT	PD-L1	PFS
NCT02274155	I	17	Locally Advanced HNSCC	MEDI6469	OX40	Safety and Feasibility
NCT04080804	II	60	Locally Advanced, Resectable HNSCC	Nivolumab + Relatlimab *vs* Nivolumab + Ipilimumab *vs* Nivolumab	PD-1, LAG-3, CTLA-4	Number of AE
NCT02955290	I/II	181	Advanced HNSCC	CIMAvax,+ Nivolumab *vs* CIMAvax + Pembrolizumab	PD-1	DLT, OS
NCT03509012	I	360	Advanced HNSCC	Durvalumab + Cisplatin + RT	PD-L1	Rate of DLT and AEs
NCT03336606	I	35	Advanced, Resectable HNSCC	MEDI0562 + Surgery	OX40	Activation of immune response
NCT04348916	I	71	Refractory, Ineligible, Relapsed HNSCC	ONCR-177 *vs* ONCR-177 + Pembrolizumab	Vaccine, PD-1	Rate of DLT and AE
NCT04129320	II/III	750	R/M HNSCC	Pembrolizumab + Chemotherapy, Enoblituzumab + MGA012, Enoblituzumab + MGA012 + Chemotherapy, MGA012 + Chemotherapy	PD-1, B7-H3, PD-1	Overall Response Rate, Incidence of AE, OS
NCT02575404	I	22	HNSCC with progression	GR-MD-02, Pembrolizumab	PD-1	Rate of AE
NCT03247712	I/II	28	HNSCC planned for surgy	Nivolumab + RT	PD-1	Rate fo Delay to Surgery
NCT04007744	I	45	R/M HNSCC	Sonidegib + Pembrolizumab	PD-1	MTD, Response rate
NCT03258554	II/III	523	Locoregionally Advanced HNSCC	Durvalumab + RT *vs* Cetuximab + RT	PD-L1	DLT, PFS, OS
NCT03083873	II	55	R/M HNSCC	LN-145/LN-145-S1	Cell transfer therapy	ORR
NCT03051906	I/II	69	Locally Advanced HNSCC	Durvalumab + Cetuximab + Radiotherapy	PD-L1	2-year PFS
NCT02892201	II	9	HNSCC with Residual Disease	Pembrolizumab	PD-1	Overall response rate
NCT03993353	II	30	R/M HNSCC	Tadalafil + Pembrolizumab	PDE-5, PD-1	Rate of DLT, OS
NCT03665285	I/II	143	Advanced, metastatic HNSCC	NC318	experimental antibody	MTD, PAD
NCT03818061	II	110	R/M HNSCC	Atezolizumab + Bevacizumab	PD-L1,	Overall response rate
NCT03228667	II	611	HNSCC with progression	ALT-803 + Pembrolizumab, Nivolumab, Atezolizumab, Avelumab	PD-1, PD-L1	ORR
NCT03319459	I	100	Advanced HNSCC	FATE-NK100	NK cell product	Incidence of DLT
NCT03633110	I/II	99	HNSCC	GEN-009 Adjuvanted Vaccine + Nivolumab, Pembrolizumab	Vaccine, PD-1	Incidence of AE, T-cell responses
NCT02376699	I	135	Advanced HNSCC	Intravenous (IV) SEA-CD40, Pembrolizumab, Subcutaneous (SC) SEA-CD40, Gemcitabine, Nab-paclitaxel	CD40, PD-1	Incidence of AE, ORR
NCT02827838	II	20	OSCC, OPSCC	Durvalumab	PD-L1	Immune effector, Immune-regulatory miR responses,
NCT04393506	I	20	Locally Advanced and Resectable OSCC	Camrelizumab, Apatinib	PD-1	Major pathologic response
NCT03673735	III	650	HPV-negative HNSCC	Durvalumab + RT+ Cisplatin *vs* Placebo + RT + Cisplatin	PD-L1	DFS
NCT03841110	I	76	Advanced HNSCC	FT500	NK cell product	Rate of DLT

This table contains only ongoing clinical trials registered on the ClinicalTrials.gov, not including terminations or completed trials.

DFS, Disease Free Survival; R/M, Recurrence/metastasis; HNSCC, Head and neck squamous cell carcinoma; ORR, Objective Response Rate; LRR, Locoregional recurrence rates; RT, Radiation therapy; PFS, Progression-free survival; SBRT, Stereotactic Body Radiotherapy; KIR, Killer cell immunoglobulin receptor(NK cell); RP2D, Recommended phase 2 dose; OS, Overall survival; pCR, Pathologic complete response; HIGRT, Hypofractionated Image-Guided Radiation Therapy; MTD, Maximum Tolerated Dose; DLTs, dose-limiting toxicities; DLT, Dose Limiting Toxicity; AE, Adverse Events; AEI, adverse events of interest; PAD, pharmacologically active dose; OSCC, Oral Squamous Cell Carcinoma; HPV , Human papillomavirus.

**Table 2:** Summary of clinical trials that have been completed.

ID	Phase	N	Inclusion criteria	Interventions	Immunotherapy targets	Primary Outcome	State
NCT02163057	I/II	22	HPV+ HNSCC	INO-3112+EP	Vaccine	Safety and Tolerability	Completed
NCT00257738	I	17	MAGE-A3+, HPV16+, R/M HNSCC	MAGE-A3, HPV16 vaccine	Vaccine	Toxicity	Completed
NCT00021424	I	20	Stage IV HNSCC	Fowlpox-TRICOM vaccine	Vaccine	Effectiveness and MTD	Completed
NCT01998542	II	12	R/M HNSCC	Cancer Vaccine (AllovaxTM)	Vaccine	Tumor Response	Completed
NCT01334177	I	13	Locally Advanced, R/M HNSCC	VTX-2337 + Cetuximab	TLR8	MTD, Toxicities	Completed
NCT00050388	II		Stage I, II, Resectable HNSCC	Allovectin-7®	Vaccine	Safety and Efficacy	Completed
NCT00843635	–	35	Resectable HNSCC	Tadalafil	PDE-5	Ratio of MDSC, T-reg Cell Tumor-specific T-cell	Completed
NCT01848834	I	297	R/M HNSCC	Pembrolizumab	PD-1	OR, OS, Rate of AE	Completed
NCT02105636	III	506	R/M HNSCC	Nivolumab, Cetuximab, Methotrexate, Docetaxel	PD-1	OS, PFS, ORR	Completed
NCT02255097	II	172	R/M HNSCC	Pembrolizumab	PD-1	ORR, Number of AE	Completed
NCT01375842	I	661	Locally Advanced, Metastatic HNSCC	Atezolizumab	PD-L1	Number of DLTs, MTD, RP2D, Rate of AE	Completed
NCT01836029	II	195	R/M HNSCC	Chemotherapy + Cet uximab + VTX-2337 *vs* Chemotherapy + Cetuximab + placebo	TLR8	PFS, OS, AE	Completed
NCT02643550	II	140	R/M HNSCC	Monalizumab + Cetuximab	NKG2A	DLTs, ORR	Completed
NCT02207530	II	112	R/M HNSCC	Durvalumab	PD-L1	ORR	Completed
NCT02426892	II	34	HPV16+, Incurable HNSCC	ISA101 + Nivolumab	Vaccine, PD-1	ORR	Completed
NCT02252042	III	495	R/M HNSCC	Pembroliziumab *vs* Active Comparator	PD-1	OS, PFS	Completed
NCT02319044	II	267	R/M HNSCC	Durvalumab, Tremelimumab, Durvalumab + Tremelimumab	PD-L1, CTLA-4	ORR	Completed

This table contains only completed clinical trials registered on the ClinicalTrials.gov.

R/M, Recurrence/metastasis; HNSCC, Head and neck squamous cell carcinoma; ORR, Objective Response Rate; PFS, Progression-free survival; RP2D, Recommended phase 2 dose; OS, Overall survival; MTD, Maximum Tolerated Dose; DLTs, Dose Limiting Toxicities; DLT, Dose Limiting Toxicity; AE, Adverse Events; HPV, Human papillomavirus; OR, Overall response.

**Figure 2:**
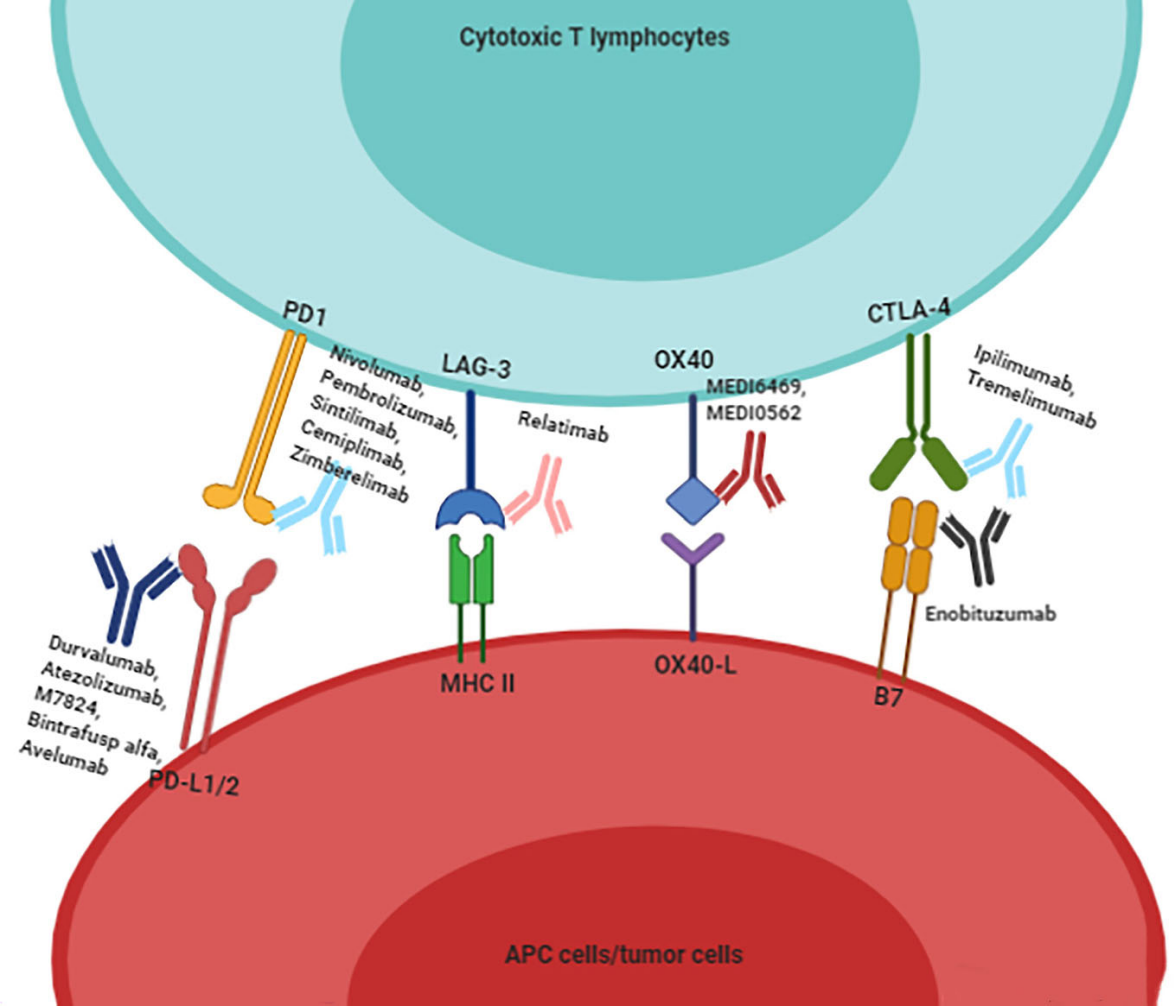
To summarize the existing immune checkpoint inhibitors for HNSCC patients. Immunotherapy drugs combine with immune checkpoints to block the inhibitory effect of immune checkpoints on cytotoxic T lymphocytes, and then activate the proliferation and infiltration of T cells.

**Figure 3:**
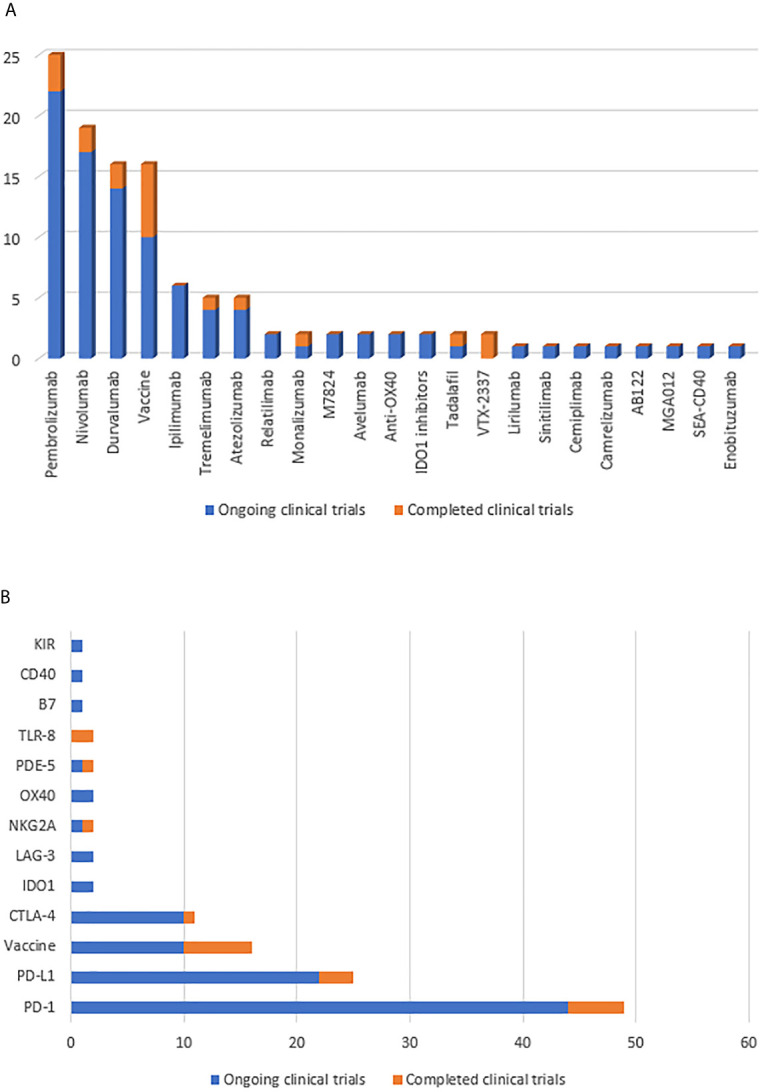
**(A)** Summarize the number of clinical trials of immunotherapy drugs for HNSCC patients. **(B)** Summarize the number of clinical trials of immunotherapy targets for HNSCC patients. Yellow indicates completed clinical trials and blue indicates ongoing clinical trials.

The number of immunotherapy trials for R/M HNSCC has been increasing. Different ICs act on different immune checkpoints; these are summarized in **Figure 2**. Great advances have been made in immunotherapy, bringing new hope for R/M HNSCC treatment. The ongoing and completed clinical trials for different drugs and targets are summarized in **Figure 3**. However, the benefits of immunotherapy are limited to a small proportion of patients with HNSCC. Therefore, it is important to identify markers that respond well to immunotherapy and further screen appropriate populations for immunotherapy (56, 57). PD-L1 has been studied as a potential biomarker in CheckMate 141, KEYNOTE 040, KEYNOTE 048 (28, 30, 58). PD-L2 has also been studied as another ligand of PD-1 (59, 60). In addition, HPV infection may be a new target to improve the efficacy of HNSCC immunotherapy.

## Immunotherapy for HPV-Associated HNSCC

In HPV-positive tumors, HPV infection leads to an increase in CRT response (61), which activates the immune response and leads to a high proportion of immune cell infiltration, thus exerting a better antitumor response (62). Conversely, HPV-negative tumors are presumed to have a relatively lower immune response. Therefore, the impact of HPV infection on the immunotherapeutic efficacy for HNSCC may be critical (63, 64).

In the KEYNOTE trials, a subset of HPV-positive patients administered pembrolizumab was evaluated to examine the relationship between HPV infection and immunotherapy. In the KEYNOTE-012, HPV-positive HNSCC patients showed a higher response rate with pembrolizumab, a higher progression-free survival for phase Ib HPV-positive tumors (4 months *vs*. 2 months), and higher objective response rate (ORR) (32% *vs*. 14%) (26, 65, 66). Similar results were also confirmed in KEYNOTE-055, although ORR and progression-free survival were similar between the two groups, overall survival in HPV-positive patients showed some advantage, even if it was not statistically significant (40). In addition, in CheckMate 141, nivolumab group showed better overall survival in HPV-positive tumors (27). A 2020 meta-analysis of 11 studies showed that HPV-positive HNSCC patients were 1.29 times more likely to respond to immunotherapy than HPV-negative HNSCC patients (risk ratio 1.29; 95% CI = 0.85-1.96; I^2^ = 0), and have a two-fold higher overall survival rate (11.5 months *vs*. 6.3 months) (29). In conclusion, the better prognosis of HPV-related HNSCC may be associated with better immunotherapy efficacy.

Given the good efficacy of immunotherapy for HPV-positive patients, a large number of immunotherapy studies targeting HPV-positive HNSCC have been conducted. Several therapeutic vaccine strategies are being studied (67). NCT00257738 is a phase I clinical trial in which patients with R/M HNSCC were administered two peptide vaccines—GL-0810 (HPV16 vaccine) and GL-0817 (MAGE-A3)—to observe their safety and immune response. The results showed that both vaccines were well tolerated and able to activate the antibody response. However, not all clinical trials have reported similar results. A phase I clinical trial using low-dose cyclophosphamide combined with the E7DNA vaccine was halted prematurely (NCT01493154).

The proportion of T cells in patients is closely related to the antitumor immune response (68). Therefore, antigen-specific T cells have become a promising immunotherapeutic strategy. An ongoing phase II clinical trial (NCT01585428II) is examining the efficacy of autologous T cell transfusion after genetic modification for the treatment of HPV-positive tumors (38). Meanwhile, A phase II clinical trial that observing the prognosis of patients with HPV-positive tumors treated with tumor-infiltrating lymphocyte (TIL) infusion was conducted (NCT01585428). MED10457 is a synthetic plasmid targeting the HPV E6/E7 antigen. Aggarwal et al. (69) found that T cells produced a large amount of HPV-specific interferon and increased antigen-specific T cell infiltration with no serious adverse reactions in HPV-positive HNSCC patients administered MEDI0457 (NCT02163057). In addition, flow cytometry showed HPV-specific PD-1-positive T cells (0% *vs*. 1.8%) after using MEDI0547. The results showed that MEDI0547 can activate the tumor immune response and generate HPV-specific T cells, which has achieved some efficacy in antitumor therapy and may be used as a supplementary treatment strategy for PD-1 inhibitors. **Table 3** summarizes ongoing clinical trials for HPV-positive HNSCC patients.

**Table 3:** Ongoing clinical trials for HPV-positive HNSCC.

ID	Phase	N	Inclusion criteria	Interventions	Immunotherapy targets	Main outcome
NCT03383094	II	114	Advanced/Intermediate-Risk p16+HNSCC	Pembrolizumab + RT *vs* Cisplatin + RT	PD-1	PFS
NCT03578406	I	20	HPV+R/M HNSCC	HPV E6-specific TCR-T cells	Engineered T cells	MTD
NCT02002182	II	15	HPV+HNSCC	ADXS 11-001 *vs* Control	Vaccine	HPV-Specific T Cell Response Rate
NCT03618134	I/II	82	HPV+OPSCC	SBRT + Durvalumab + surgery *vs* SBRT + Durvalumab + Tremelimumab + surgery	PD-L1, CTLA-4	Incidence of adverse events, PFS
NCT04260126	II	96	HPV16+R/M HNSCC	Pembrolizumab+PDS0101	PD-1, T-cell immunotherapy	ORR
NCT03162224	I/II	35	HPV+R/M HNSCC	MEDI0457, Durvalumab	Vaccine, PD-L1	Number of patients with changes in ECG, Occurrence of SAEs

This table contains only clinical trials registered on the ClinicalTrials,gov for HPV-positive HNSCC that are in progressing and do not include terminated or completed.

RT, Radiation therapy; HNSCC, Head and neck squamous cell carcinoma; PFS, Progression-free survival; R/M, Recurrence/metastasis; MTD, The Maximum Tolerated Dose; OPSCC, Oropharyngeal squamous cell carcinoma; SBRT, Stereotactic Body Radiotherapy; ORR, Objective Response Rate; ECG, Electrocariogram; SAEs, Serious adverse events; HPV, Human papillomavirus.

In conclusion, immunotherapy for HPV-positive tumors may have better effect. Next, we will further review the molecular mechanisms by which HPV-related HNSCC has a better immunotherapeutic effect.

## Immune Characteristics of HPV-Positive Tumors

The differences in the efficacy of immunotherapy are mainly related to the intrinsic characteristics of HPV-positive tumors, including tumor immunogenicity, immune cell infiltration, and IC expression (70).

In 2013, differences in immune cell infiltration between HPV-positive and HPV-negative HNSCC were reported for the first time; higher proportions of B cell infiltration were noted in HPV-positive tissues (71). Subsequent research by Wood et al. confirmed this finding (72). Studies have confirmed a larger proportion of CD8+T cell infiltration in HPV-positive HNSCC (31, 73, 74), which is strongly associated with improved prognosis of HPV-positive tumors (75, 76). TILs are often present in tumor tissues and represent an adaptive host antitumor response. Chakravarthy et al. (77) measured CD4 and CD8 mRNA abundance to compare TIL levels between HPV-positive and HPV-negative tumors, showing higher TIL levels in the HPV-positive group. Significant increases in multiple T cell markers were also found, indicating that lymphocyte infiltration may be a key factor leading to survival differences in HPV-positive HNSCC (77). Subsequently, in a 2014 study (78), patients were divided into high-risk and low-risk groups based on TIL levels. The results showed a significantly higher 3-year survival rate in the high-TIL group than in the low-TIL group. Similar results were reported in two studies by King and Nasman (79, 80). Therefore, a high proportion of TILs may play an antitumor role through adaptive host immune response, which may contribute to increased survival of HPV-positive patients (81, 82). Treg cells are a subset of T cells with immunosuppressive effects, which inhibit the immune responses of other cells through Foxp3, CD25, and other transfer factors. Treg cells are usually associated with poor prognosis. However, recent findings in HPV-positive HNSCC show the opposite (72). NK cells are an important part of innate immunity. In HPV-positive tumors, NK cells also have higher levels of infiltration (83) and are associated with better prognosis (31, 72). Therefore, in HPV-positive tumor tissues, the high proportions of TILs infiltration, CD8+ T cells, Treg cells, NK cells, and other immune cells may explain the better prognosis (84–86).

In addition, in an analysis of TCGA Database, Marij et al. (87) found that HPV-specific T cells are present in HPV-positive tumors and that their presence is associated with better survival. Studies have also confirmed the presence of HPV-specific T lymphocytes in the peripheral blood of HPV-positive HNSCC patients *in vitro* (88). A recent study compared the immune spectra of HPV-positive and HPV-negative tumors by single-cell RNA and multispectral immunofluorescence analysis, reporting the presence of germinal center B cells in HPV-positive TILs and low percentages of B cells in HPV-negative HNSCC, with most in a non-germinal center state (89). Therefore, differences in immune cell infiltration may be one of the reasons for the good prognosis in HPV-positive HNSCC.

Through gene expression analysis, Wood et al. confirmed increased expressions of PD1, CTLA-4, and T-cell immunoglobulin and mucin-domain containing-3 (TIM-3) in HPV-positive HNSCC (72, 90). With the discovery of PD-L1 in head and neck tissue, higher PD-L1 expression and activity have been found in HPV-driven HNSCC (91, 92), which was associated with a good prognosis (93). PD1+ TILs were present in HPV-positive HNSCC, suggesting that PD-1 may play an important role in HPV-positive HNSCC (91). Some studies also speculated that TILs promote PD-L1 expression in HPV-positive HNSCC tumor cells through the secretion of pheromone -γ (81) to explain the expression levels exceeding 70% (91, 94). The higher expression level of CTLA-4 may be related to the higher proportion of T cells expressing CTLA-4 (31). During HPV infection, high levels and activity of PD-1, PD-L1, and CTLA-4 may contribute to the good effects of ICs in HPV-positive HNSCC patients.

## Conclusion

After surgery, radiotherapy, chemotherapy, and targeted therapy, the emergence of immunotherapy has ushered in a new era in the treatment of HNSCC. Inhibitors based on immune checkpoints such as PD-1, PD-L1 have achieved good clinical efficacy, and clinical studies on multitarget combined immunotherapy, immunotherapy combined with traditional radiotherapy, chemotherapy, and IC combined with small molecular agonists are also being performed. The development of immunotherapy brings hope of new treatment options and survival for advanced HNSCC patients. However, their efficiency is only about 20%. Therefore, patient selection and the discovery of effective biomarkers for immunotherapy are important.

HPV infection is a newly discovered risk factor of HNSCC. Patients with HPV-positive tumors have better prognosis than HPV-negative tumors, which may be related to better treatment outcomes. Subgroup analysis of the KEYNOTE-012, 055 trials showed better responses in HPV-positive patients using pembrolizumab. Subsequently, trials on immunotherapy for HPV-positive HNSCC patients were gradually performed, including those for a immunomodulatory vaccine for HPVE6/E7 and auto-specific T-cell transfusion. HPV-positive patients have become a special group for immunotherapy. However, some studies have suggested that there is no significant advantage in immunotherapy for patients with HPV-positive HNSCC (95). So further research is needed to determine if immunotherapy can achieve better clinical benefits in these patients.

Studies have confirmed higher immune cell infiltration and expression levels of PD-1, PD-L1, and other immune checkpoints in HPV-positive tumors, which may be the key factor for better efficacy of immunotherapy. Further research on the immune system and immune microenvironment of HPV-positive tumors will provide a new theoretical basis for individual immunotherapy of HPV-positive patients and lay a foundation for screening suitable populations for immunotherapy. In addition, further *in vitro* and *in vivo* studies of HPV-positive tumors related to the immune system and further exploration of potential immune-related targets in HPV-positive cells are essential to improve the immunotherapeutic efficacy of immune-tolerant HNSCC.

## Author Contributions

Conceptualization, XJ and YX. Software, investigation, HW, QZhang, YYZ, ZZ, and ZL. Resources, YX. Writing-original draft preparation, HW, QZhao, SL, ZL, and XJ. Writing-review and editing, LM, YX, and XJ. Funding acquisition, XJ. All authors contributed to the article and approved the submitted version.

## Funding

This research was funded in part by grants from the National Natural Science Foundation of China (81570344, to YX), the Norman Bethune Program of Jilin University (2015225, to YX and 2015203, to XJ), National Key R&D Program of China (2017YFC0112100, to XJ), and the Jilin Provincial Science and Technology Foundations (20180414039GH to YX and 20190201200JC to XJ).

## Conflict of Interest

The authors declare that the research was conducted without any commercial or financial relationships that could be construed as a potential conflict of interest.
